# Impaired Photic Entrainment of Spontaneous Locomotor Activity in Mice Overexpressing Human Mutant α-Synuclein

**DOI:** 10.3390/ijms19061651

**Published:** 2018-06-03

**Authors:** Martina Pfeffer, Zuzana Zimmermann, Suzana Gispert, Georg Auburger, Horst-Werner Korf, Charlotte von Gall

**Affiliations:** 1 Institut für Anatomie II, Fachbereich Medizin, Heinrich Heine Universität, Universitätsstr. 1, D-40225 Düsseldorf, Germany; Charlotte.Gall@med.uni-duesseldorf.de; 2 Dr. Senckenbergische Anatomie II, Fachbereich Medizin, Goethe-Universität Frankfurt, Theodor-Stern-Kai 7, D-60590 Frankfurt am Main, Germany; Zuzana.Zimmermann@gmx.de; 3 Experimental Neurology, Department of Neurology, Goethe-Universität Frankfurt, Theodor-Stern-Kai 7, D-60590 Frankfurt am Main, Germany; Gispert-sanchez@em.uni-frankfurt.de (S.G.); Auburger@em.uni-frankfurt.de (G.A.); 4 Institut für Anatomie I, Fachbereich Medizin, Heinrich Heine Universität, Universitätsstr. 1, D-40225 Düsseldorf, Germany; Korf@uni-duesseldorf.de

**Keywords:** Parkinson disease, endogenous clock, vesicular glutamate transporter 2, actography, circadian rhythms

## Abstract

Parkinson’s disease (PD) is characterized by distinct motor and non-motor symptoms. Sleep disorders are the most frequent and challenging non-motor symptoms in PD patients, and there is growing evidence that they are a consequence of disruptions within the circadian system. PD is characterized by a progressive degeneration of the dorsal vagal nucleus and midbrain dopaminergic neurons together with an imbalance of many other neurotransmitters. Mutations in α-synuclein (SNCA), a protein modulating SNARE complex-dependent neurotransmission, trigger dominantly inherited PD variants and sporadic cases of PD. The A53T SNCA missense mutation is associated with an autosomal dominant early-onset familial PD. To test whether this missense mutation affects the circadian system, we analyzed the spontaneous locomotor behavior of non-transgenic wildtype mice and transgenic mice overexpressing mutant human A53T α-synuclein (A53T). The mice were subjected to entrained- and free-running conditions as well as to experimental jet lag. Furthermore, the vesicular glutamate transporter 2 (VGLUT2) in the suprachiasmatic nucleus (SCN) was analyzed by immunohistochemistry. Free-running circadian rhythm and, thus, circadian rhythm generation, were not affected in A53T mice. A53T mice entrained to the light–dark cycle, however, with an advanced phase angle of 2.65 ± 0.5 h before lights off. Moreover, re-entrainment after experimental jet lag was impaired in A53T mice. Finally, VGLUT2 immunoreaction was reduced in the SCN of A53T mice. These data suggest an impaired light entrainment of the circadian system in A53T mice.

## 1. Introduction

The sleep–wake cycle is generated by a switch between sleep-promoting and -arousing brain regions under the control of the circadian rhythm generator in the suprachiasmatic nucleus (SCN) [1]. The SCN generates circadian rhythms which are entrained to the rhythmically occurring episodes of light and darkness in the environment by photic information from the retina. SCN outputs convey temporal information about the environmental light conditions to the rest of the brain and body, thereby regulating the sleep wake cycle and other physiological functions [2]. A misalignment of the sleep patterns with the environment characterizes circadian rhythm sleep disorders [3,4] Sleep disturbances are a major and early symptom of patients with neurodegenerative disorders such as Parkinson’s disease (PD), dementia with Lewy bodies, and multiple system atrophy (reviewed by [5,6,7,8]). These diseases are characterized by abnormal accumulation of α-synuclein (SNCA) aggregates in the brain and are thus called synucleinopathies. In patients with PD, especially the timing of sleep is affected [9], as they show daytime sleepiness [10] and nighttime insomnia [11,12,13,14], which has a great impact on their quality of life.

The etiology of this sleep disorders in PD is multifactorial, related to alterations in dopamine signaling and neurodegeneration in various brain regions, not only in the brain stem [15] but also in the striatum [16], the thalamus [17], as well as the sleep arousing orexin neurons of the lateral hypothalamus [18,19,20]. The occurrence of excessive daytime sleepiness in PD patients is linked to reductions in hypothalamic dopamine D3 receptor availability [18]. Moreover, we could show earlier that in a mouse model of neurodegeneration, a disturbed sleep–wake cycle rhythm was associated with an impairment of circadian light perception and structural changes in the lateral hypothalamus [21]. These and many other studies suggest that both sleep homeostasis as well as sleep timing under the control of the circadian system contribute to the pathophysiology of sleep disorders in PD.

Polymorphic variants in the *SNCA* gene have been associated with susceptibility to idiopathic PD [22], and missense mutations as well as multiplications of the *SNCA* gene have been shown to be responsible for familial forms of PD [23,24,25]. Under physiological conditions, SNCA is abundantly expressed in neurons. SNCA is a presynaptic protein primarily associated with synaptic vesicles and plays a role in signaling, neurotransmitter release, and synaptic maintenance [26,27,28,29]. However, the role of abnormal SNCA in the development of circadian rhythm sleep disorders is still enigmatic. The best animal models for synucleinopathies are transgenic mice overexpressing regular human SNCA [30] or human SNCA with a point mutation (A53T; [31,32,33,34,35]) which has been linked to familial autosomal dominant early-onset PD [31].

Transgenic mice overexpressing regular human SNCA show reduced wheel running activity, impaired entrainment of locomotor activity to a skeleton photoperiod, and a slightly changed pattern in sleep behavior [36]. However, the impact of the A53T missense mutation on the entrainment of circadian rhythms is still unknown. Therefore, we analyzed the entrainment of circadian rhythms in spontaneous locomotor activity in A53T mice. Furthermore, we analyzed vesicular glutamate transporter 2 (VGLUT2), as a maker for glutamatergic input from the retina to the SCN, by immunohistochemistry. A53T mice did not show a difference in the circadian rhythms of spontaneous locomotor activity under constant darkness, indicating an intact circadian rhythm generator. A53T mice entrained to a light–dark cycle, however, with a significant advance of the activity onset relative to the onset of darkness. In addition, A53T mice showed a significantly less stably entrained rhythm, especially after experimental jet lag. These data suggest an impaired light entrainment of the circadian system. Moreover, VGLUT2 immunoreaction in the SCN of A53T mice was reduced. These data suggest an impaired photic entrainment in A53T, presumably as a consequence of changes in glutamatergic neurotransmission between the retina and the SCN.

## 2. Results

### 2.1. SNCA Immunoreaction Is Increased in the SCN of A53T Mice

**Figure 1:**
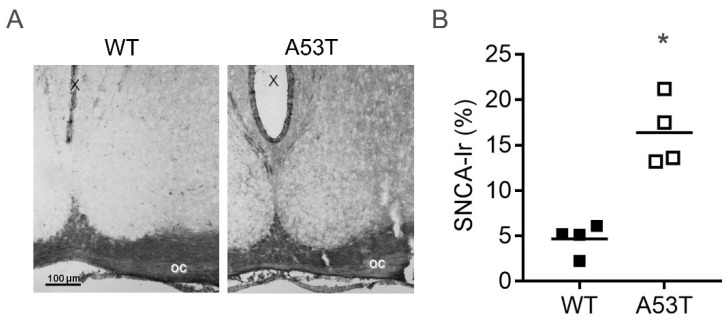
α-Synuclein (SNCA) immunoreaction (Ir) in the suprachiasmatic nucleus (SCN) of wild-type (WT) and A53T-SNCA mice. (**A**) Representative microphotographs of SNCA Ir in the SCN of WT and A53T mice; oc, optic chiasm, X, third ventricle, Bar = 100 µm; (**B**) Semiquantitative analysis of SNCA Ir in the SCN of WT and A53T mice. Data are shown as the area of SNCA Ir in % of the SCN area; *: *p* < 0.05, Mann–Whitney test.

In wild-type (WT) mice, SNCA immunoreaction in the SCN was very low. In A53T mice, SNCA immunoreaction showed a punctate staining in the neuropil (Figure 1A), and the area of SNCA immunoreaction in the SCN was significantly larger than in WT mice (Figure 1B).

### 2.2. Rhythmic Spontaneous Locomotor Activity under Constant Darkness Is Not Affected in A35T Mice

**Figure 2:**
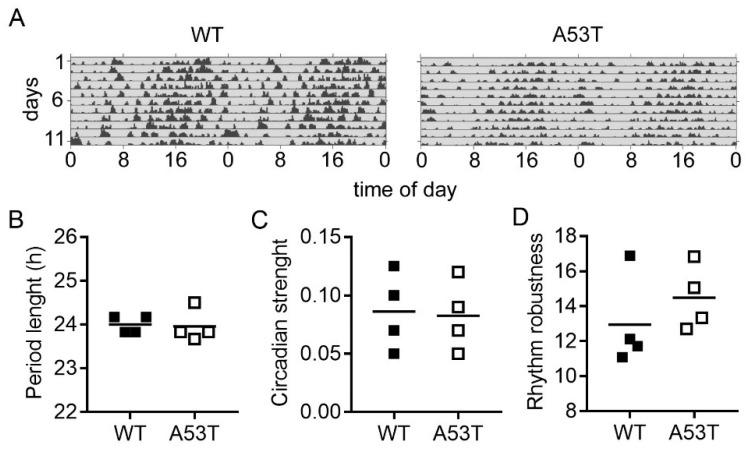
Analysis of locomotor activity and rhythm robustness in constant darkness. (**A**) Representative double-plotted actograms of spontaneous locomotor activity of WT and A53T mice in constant darkness. Grey shading indicates darkness; (**B**) Major peak in the period length based on Chi-square periodograms; (**C**) Circadian strength based on Fast Fourier analysis; (**D**) Rhythm robustness based on Qp analysis. There were no significant differences (Mann–Whitney test) in any of these parameters between WT and A53T mice.

In constant darkness, both genotypes showed a free-running circadian rhythm of spontaneous locomotor activity (Figure 2A). Chi-squared periodograms revealed a period length of the locomotor activity rhythms of 24 ± 0.09 h in WT mice and of 23.96 ± 0.18 h in A53T mice (Figure 2B). Circadian strength (Figure 2C) and rhythm robustness (Figure 2D) were not significantly different between the two genotypes.

### 2.3. A35T Mice Show a Higher Daytime Activity and an Advanced Phase Angle of Entrainment under LD 12:12 Conditions

**Figure 3:**
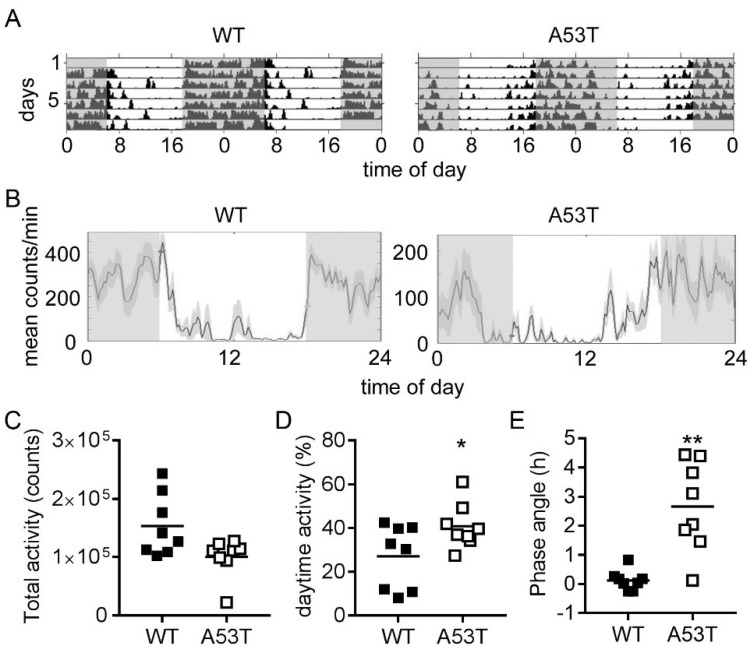
Analysis of locomotor activity in a 12 h light–12 h dark (LD 12:12) photoperiod. (**A**) Double-plotted actograms of spontaneous locomotor activity in LD 12:12 from representative WT and A53T mice. Grey shading indicates periods of darkness; (**B**) Representative cumulative activity profiles of WT and A53T mice under LD 12:12 conditions. Grey shading indicates periods of darkness. Note that the onset activity in the WT mouse is tightly coupled to the onset of the dark phase, whereas the A53T mouse becomes active during the second half of the light period. Graphs show the (**C**) total activity, (**D**) daytime activity, and (**E**) phase angle of entrainment. A positive phase angle indicates onset of activity prior to lights off; *n* = 8 mice per genotype; *: *p* < 0.05, **: *p* < 0.01, Mann–Whitney test.

WT and A53T mice showed synchronization of spontaneous locomotor activity to the light/dark 12:12 cycle (LD 12:12), with lower activity during the light phase and higher activity during the dark phase (Figure 3A,B). A53T mice showed a slightly reduced total amount of spontaneous locomotor activity compared to WT mice (Figure 3C). However, daytime activity was significantly higher in A53T mice compared to WT mice (Figure 3B,D). In WT mice, activity onset was tightly coupled to lights off, whereas, in A53T mice, activity onset occurred considerably before lights off (Figure 3A,B). The phase angle of entrainment was significantly larger in A53T mice (2.65 ± 0.54 h) compared to WT mice (0.12 ± 0.12 h) (Figure 3E).

### 2.4. Re-Entrainment after Experimental Jet Lag Is Affected in A53T Mice

**Figure 4:**
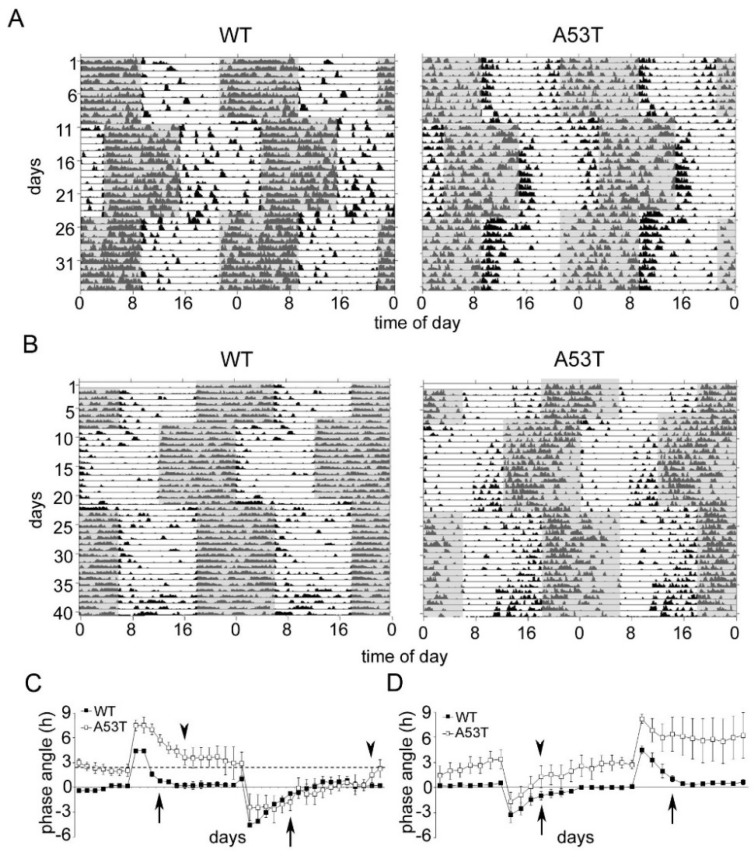
Re-entrainment of spontaneous locomotor activity rhythms in WT and A53T mice subjected to experimental jet lag. (**A**) Double-plotted actograms of locomotor activity from representative WT and A53T mice subjected to a 6 h phase delay followed by a 6 h phase advance back to the former photoschedule. Grey shading indicates periods of darkness; (**B**) Double-plotted actograms of locomotor activity from representative WT and A53T mice subjected to a 6 h phase advance followed by a 6 h phase delay back to the former photoschedule. Grey shading indicates periods of darkness; (**C**) Phase angle of locomotor activity in WT and A53T mice after the 6 h phase delay followed by the 6 h phase advance. The data are expressed as the mean ± SEM of four mice in each genotype. Arrow and arrowheads indicate the first day of re-entrainment to the new photoschedule in WT and A53T mice, respectively. Re-entrainment to a new photoschedule is defined by the difference between phase angle and basal phase angle <1.5 h. In WT mice, the basal phase angle is very close to the *x*-axis; in A53T mice, the basal phase angle is indicated by the dashed line; (**D**) Phase angle of locomotor activity in WT and A53T mice after the 6 h phase advance followed by the 6 h phase delay. The data are expressed as the mean ± SEM of four mice in each genotype. Arrow and arrowheads indicate the first day of re-entrainment to the new photoschedule in WT and A53T mice, respectively. Data are expressed as the mean ± SEM; *n* = 8 mice in each experimental group, *n* = 4 mice per genotype in each experimental group.

In the experimental jet lag group, which was first subjected to a phase delay, WT mice re-entrained to the phase delay within four days and to the phase advance within 6 days, whereas the A53T mice needed 7 days for re-entrainment after the delay and 16 days for the phase advance (Figure 4A,C). In the experimental jet lag group which was first subjected to a phase advance, WT mice re-entrained to both the phase advance and the phase delay within 4 days (Figure 4B,D). A53T mice also needed 4 days for re-entrainment after the advance (Figure 4B,D). However, after the phase delay, back to the former standard photoperiod, A53T mice responded very heterogeneously: two mice re-entrained within 4 days, whereas two mice did not re-entrain within the observation period (Figure 4A,C, Supplemental Figure S1).

### 2.5. Vesicular Glutamate Transporter Type 2 Immunoreaction Is Reduced in A53T-SNCA Mice

**Figure 5:**
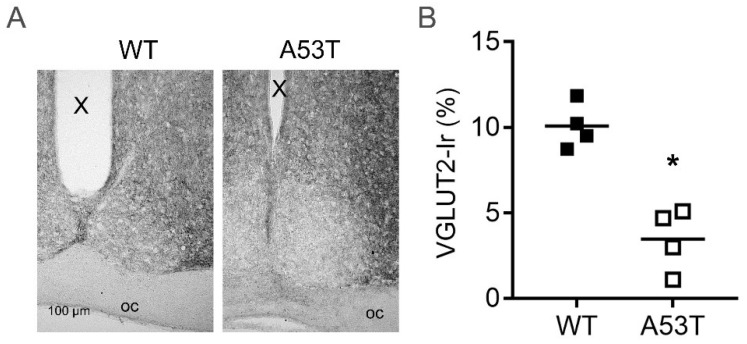
VGLUT2 immunoreaction (Ir) in the SCN of WT and A53T mice. (**A**) Representative photomicrographs of VGLUT2 Ir in the SCN of WT and A53T-SNCA mice; oc, optic chiasm, X, third ventricle; (**B**) The graph shows a semiquantitative analysis of VGLUT2 Ir in the SCN of WT and A53T mice. The data are shown as the area of vGLUT2 Ir in % of the SCN area; *n* = 4 mice for each genotype; *: *p* < 0.05 (Mann–Whitney test).

As glutamate is an important neurotransmitter in transmitting light information from the retina to the SCN, we analyzed VGLUT2 immunoreaction. In A53T mice, VGLUT2 immunoreaction in the SCN was significantly reduced compared to WT animals (*p* < 0.05, Figure 5).

## 3. Discussion

Many neurodegenerative disorders, including Parkinson’s disease (PD), are associated with sleep disturbances [37], which are manifested by disorganized locomotor activity patterns in mice and humans [21,38,39,40]. In this study, we analyzed the rhythmic spontaneous locomotor activity in transgenic mice expressing mutant human A53T α-synuclein, which is associated with inherited early-onset PD in humans.

SNCA immunoreaction in the SCN was increased in A53T mice compared to WT mice, presumably because of an accumulation of mutant A53T SNCA, as the antibody detects both the endogenous and the mutant forms. A53T mice kept in constant darkness showed a circadian rhythm in spontaneous locomotor activity with the same period length and robustness as WT mice. This demonstrates the integrity of the molecular clockwork within the SCN and is in accordance with results from SNCA-overexpressing mice, which also did not show alterations in the period length and rhythmicity of wheel running activity in constant darkness [36]. Thus, overexpression of regular SNCA or of the human mutant A53T-SNCA does not affect endogenous circadian rhythm generation.

The total amount of spontaneous locomotor activity was slightly reduced in A53T mice compared to WT mice. This is consistent with the reported progressive reduction of spontaneous vertical movement and activity which can be observed in A53T mice from the age of 6 months onward [41] and with the age-dependent decrease in wheel running activity in SNCA-overexpressing mice [36]. Spontaneous locomotor activity in A53T mice was synchronized to the LD 12:12 regime, thus they were able to entrain. This suggests that melanopsin-expressing intrinsically photoreceptive retinal ganglion cells, which are essential for photoentrainment of the circadian system [42,43,44], were probably not affected. However, activity during the light phase (daytime activity) was significantly increased in A53T mice compared to WT mice. In A53T mice, daytime activity occurred predominantly during the second half of the light phase, thus with an advanced phase angle. This suggests that the inhibition of activity by light (masking) as well as the photic entrainment of circadian rhythms were impaired in A35T mice. The molecular mechanisms of masking are largely unknown, but photic entrainment involves the activation of cFOS [45], the phosphorylation of CREB [46,47,48], and the expression of Per1 [48]. A major regulator of photic entrainment is glutamate, released from intrinsically photoreceptive retinal ganglion cells (iRGCs) to the SCN [49]. The glutamatergic neurotransmission in the retina relies on the storage of glutamate in synaptic vesicles by the vesicular glutamate transporters VGLUT1 and VGLUT2 [50,51,52]. Importantly, mice lacking VGLUT2 specifically in iRGCs also show an advanced phase angle of entrainment with a comparable magnitude (Opn4^CRE/+^; VGLUT^flox/flox^ mice) [53] and impaired masking (Vglut2-cKO) [54]. We found a significant reduction of VGLUT2 immunoreaction in the SCN of A53T mice, suggesting that the advanced phase angle and impaired masking might be a consequence of impaired glutamatergic neurotransmission from the iRGCs to the SCN. α-Synuclein has been shown to modulate neurotransmitter vesicle dynamics in the presynaptic compartment by acting as a chaperone for the SNARE complex [55]. A53T mice showed a progressive dysfunctional neurotransmission and impaired synaptic plasticity [33,34] as well as a dysregulation of the 14-3-3 chaperone [56]. Thus, the impaired responses to light in A53T mice could be attributed to a disturbed light input into the circadian system. Still, it cannot be ruled out that the advanced phase angle of spontaneous locomotor activity in A53T mice is attributable to disturbed circadian clock output signaling as, e.g., mice with a targeted deletion of the clock output gene prokineticin 2 also show an increase in daytime locomotor activity [57]. This may confer variability in the timing of a circadian clock output signal relative to the oscillator itself. Also, dopaminergic influence in the light adaptation cannot be excluded, since, in the retina, dopamine has been shown to modulate light input to the SCN [9,58], and, in flies, α-synuclein mutation in serotonergic and dopaminergic neurons results in abnormal sleep-like behavior, altered locomotor activity, and abnormal circadian periodicity [59].

In the jet lag experiments, phase delays were associated with a lengthening of the dark phase, and thus light, during the first half of the former dark phase, whereas phase advances were associated with a shortening of the light phase, and thus darkness, during the first half of the former light phase. As nocturnal light is the strongest signal for re-entrainment of the circadian system to a phase shift [49], a phase advance is more challenging than a phase delay [60]. In the experimental jet lag group, which was subjected to the phase delay before the phase advance, A53T mice needed on average three and 10 days longer than WT mice to re-entrain after the delay and advance, respectively. This is consistent with the hypothesis of a disturbed light input into the circadian system. However, in the experimental jet lag group, which was subjected to the advance before the delay, A53T mice re-entrained as fast as WT mice after the advance, whereas the response to the delay was very heterogeneous, and some mice did not re-entrain within the entire observation period (20 days). As Opn4^CRE/+^; VGLUT^flox/flox^ mice can cope with this kind of experimental jet lag [53], the perturbation of re-entrainment in A53T is presumably not only an effect of impaired glutamatergic signaling. Retinal dopamine is reduced in PD patients and plays an important role in light adaptation [58]. Also, the distinct heterogeneity in time needed for re-entrainment after the phase delay observed in A53T mice could be attributable to genetic polymorphisms in clock-controlled genes and to light-sensitive modulators of gene expression such as miRNAs [61]. Therefore, studies with larger sampling and analyses of retinal neurotransmission, polymorphisms in clock-controlled genes, and miRNAs are needed. Furthermore, a dark signal during the light phase is an additional resetting signal for the SCN circadian clock by a PACAP–cAMP-dependent mechanism [62,63]. In addition, nocturnal light not only is a strong signal for re-entrainment but also can disturb the circadian system resulting in a variety of health problems [64]. Thus, the inability of some A53T mice to re-entrain to the phase delay after a phase advance could be a consequence of an impaired PACAP–cAMP-signal transduction and/or of a disruption of the circadian system. Moreover, age might also be an important factor, as A53T mice showed not only a progressive dysregulation of synaptic function [33] but also an age-dependent astrogliosis [56], and astrocytes play an important role in circadian timekeeping via glutamatergic signaling [65].

In summary, we could show that mice overexpressing human mutant A53T-SNCA displayed impaired masking, entrainment, and re-entrainment after experimental jet lag. This was associated with a decrease in VGLUT2 immunoreaction in the SCN, suggesting an affected glutamatergic signaling of retinal ganglion cells, providing the input into the circadian system. Our study might help to enlighten the pathomechanisms of sleep–wake disturbances in PD patients.

## 4. Materials and Methods

### 4.1. Animals

All experiments with animals reported in this manuscript were conducted in accordance with the policy on the use of Animals in Neuroscience Research and the Policy on Ethics as approved by the Society for Neuroscience and by the European Communities Council Directive ((89/609)EEC) in 1986 and the Regierungspräsidium Darmstadt (Gen.Nr.F6/19).

Transgenic mice (FVB/N) expressing the Parkinson disease-specific mutation α-synuclein Ala53Thr (A53T) were generated and characterized as previously described [41]. We used the homozygous PrPmtB mouse line with high overexpression levels of Ala53Thr-α-synuclein under the control of the murine neuron-specific prion protein promotor (PrP Genbank # U52821) [41]. The A53T mice expressing the mutant human α-synuclein were compared with non-transgenic FVB/N (WT) mice.

Male A53T and WT mice (4–6 month of age) were obtained by breeding heterozygous mice in the FELASA-certified Central Animal Facility (ZFE) of the Goethe University Medical Faculty, Frankfurt am Main, under routine health monitoring. The genotype was confirmed by PCR with tail biopsy DNA. A total of 16 mice of each strain were adapted to a standard photoperiod of 12 h light–12 h dark (LD 12:12) for at least 10 days before the start of the experiments, with access to food and water ad libitum. Light intensity was 44 µW/cm^2^ during the light phase and <5 µW/cm^2^ (dim red light) during the dark phase.

### 4.2. Experimental Jet Lag

A53T and WT mice were housed in individual cages equipped with infrared movement detectors linked to an automated recording system (Mouse-E-Motion, Hamburg, Germany) to monitor spontaneous locomotor activity, as described [66]. For the jet lag experiments, one set of mice (four for each genotype) were subjected to an acute delay of the light–dark cycle by 6 h (delayed lights off). After 14 days, the light–dark cycle was advanced by 6 h, back to the former standard photoperiod. A second set of mice (four for each genotype) were subjected to a reversed experimental jet lag: first a 6 h advance of the LD cycle and, after 14 days, a 6 h delay of the LD cycle.

The number of animals used in this experiment was *n* = 16 (eight mice for each genotype).

### 4.3. Data Analysis of Locomotor Activity Rhythms

Spontaneous locomotor activity was continuously recorded in 10 min intervals with infrared movement detectors linked to an automated recording system (Mouse-E-Motion, Hamburg, Germany) as described [60]. The same detectors simultaneously recorded light exposure. To activate the detector, the mouse needs to move at least half of its body length.

Actograms, period length (tau), activity onset calculation, χ^2^ periodogram analysis, Fast Fourier Transform (FFT) analysis, and activity profiles with acrophases were generated using Clocklab software (Actimetrics, Wilmette, IL, USA) as described [60,67]. Overall activity, relative daytime activity, circadian strength, rhythm robustness, period length were calculated on the basis of observation periods of 12 consecutive days in LD 12:12 and/or in dark–dark (DD).

The circadian strength was defined by FFT analysis at the peak within the range of 21–28 h. For χ^2^ analysis, the *p*-value was set to *p* < 0.05. Rhythm robustness was measured by the Qp-value of the χ^2^ periodogram analysis ([68]; http://www.circadian.org/periodogram.html). Activity onsets were detected by automated onset time calculation provided by ClockLab, as described [60]. The phase angle of activity onset to lights off was calculated by the difference in the time between lights off and the onset of activity. The onset of activity prior to lights off was expressed as a positive phase angle. The basal phase angle of entrainment was analysed in naïve mice which did not receive any phase shifts on the basis of an observation period of 12 consecutive days in LD. Re-entrainment to a new photoschedule after experimental jet lag was defined by the difference between phase angle and basal phase angle <1.5 h.

### 4.4. Immunohistochemistry

For immunocytochemistry, four mice of each genotype, kept in a standard photoperiod, were sacrificed. The anesthetized mice (100 mg ketamine/kg bodyweight and 10 mg xylazine/kg bodyweight, i.p.) were perfused transcardially with a sodium chloride solution (0.9%), followed by 4% paraformaldehyde in 0.02 M phosphate-buffered saline (PBS). The brains were dissected, postfixed in 4% paraformaldehyde overnight at 4 °C, and cryoprotected in 20% sucrose. The brains of A53T-SNCA and WT mice were cut in a cryostat into coronal 16 µm-thick sections and frozen at −20 °C until further use.

Immunocytochemistry was performed as described [66]. Nonspecific labeling was reduced by incubating the sections in PBS, containing 0.3% Triton, 1% bovine serum albumin, and 2% normal goat serum. Subsequently, the sections were incubated overnight with antibodies against α-synuclein (1:500; BD Transduction Laboratories; Los Angeles, CA, USA) or against the vesicular glutamate transporter type 2 (VGLUT2, 1:2000; Merck Millipore; Darmstadt, Germany). The primary antibodies employed in this study were characterized in previous studies [69,70,71]. Immunoreactions were visualized with a standard ExtrAvidin–biotin labeling method (Vector Laboratories, Peterborough, UK), using 0.05% 3.3′-diaminobenzidine (DAB) as the chromogen, as described previously [46,72]. The slides were coverslipped, and microphotographs were taken for quantitative analyses using a Zeiss Axioplan microscope at 200× magnification. Thereafter, the coverslips were removed, and the sections were counterstained with haematoxylin. Microphotographs were taken for qualitative analyses.

### 4.5. Quantitative Analyses of Immunohistochemistry

A semiquantitative analysis of the immunoreactions was performed by use of NIH ImageJ software (NIH; Bethesda, MD, USA). The immunoreactions in the SCN were determined by an observer blind to the genotype of the animals. The digitalized images were converted into 8-bit grey scale before image segmentation. The SCN region was selected using the freehand draw tool. The threshold was kept constant for all analyzed sections. The area of immunoreaction above the threshold and the area of the SCN were determined in two representative sections through the SCN of each animal and averaged. The data are expressed as percent of immunoreaction of total SCN area.

### 4.6. Statistical Analyses

Statistical analyses were performed with Graph Pad Prism (San Diego, CA, USA). All data are presented as the mean ± SEM of eight (behavioral experiments) or four (immunohistochemistry) animals/genotype. Differences between groups were determined by the Mann–Whitney test. Values were considered significantly different with *p* < 0.05.

## Supplementary Materials

Supplementary materials can be found at http://www.mdpi.com/1422-0067/19/6/1651/s1.
