# Supplementary figures and images for: Impaired Photic Entrainment of Spontaneous Locomotor Activity in Mice Overexpressing Human Mutant α-Synuclein
Source: Int J Mol Sci. 2018 Jun 3;19(6):1651. doi: 10.3390/ijms19061651 (PMC6032049; doi:10.3390/ijms19061651)

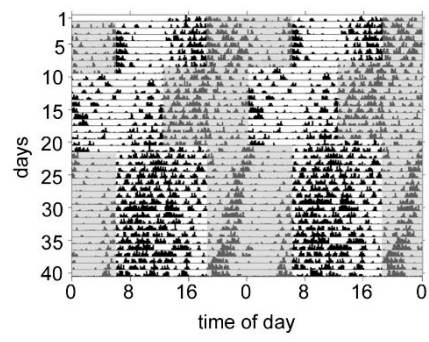

Figure S1.

Supplement: Supplementary file 1 [file ijms-19-01651-s001.pdf]
